# A comparison on effects of normalisations in the detection of differentially expressed genes

**DOI:** 10.1186/1471-2105-10-61

**Published:** 2009-02-13

**Authors:** Monica Chiogna, Maria Sofia Massa, Davide Risso, Chiara Romualdi

**Affiliations:** 1 Department of Statistical Sciences, University of Padova, via C. Battisti 241, 35121 Padova, Italy; 2 CRIBI biotechnology Center, University of Padova, via U. Bassi 58/B, 35121 Padova, Italy

## Abstract

**Background:**

Various normalisation techniques have been developed in the context of microarray analysis to try to correct expression measurements for experimental bias and random fluctuations. Major techniques include: total intensity normalisation; intensity dependent normalisation; and variance stabilising normalisation. The aim of this paper is to discuss the impact of normalisation techniques for two-channel array technology on the process of identification of differentially expressed genes.

**Results:**

Through three precise simulation plans, we quantify the impact of normalisations: (a) on the sensitivity and specificity of a specified test statistic for the identification of deregulated genes, (b) on the gene ranking induced by the statistic.

**Conclusion:**

Although we found a limited difference of sensitivities and specificities for the test after each normalisation, the study highlights a strong impact in terms of gene ranking agreement, resulting in different levels of agreement between competing normalisations. However, we show that the combination of two normalisations, such as glog and lowess, that handle different aspects of microarray data, is able to outperform other individual techniques.

## 1 Background

Microarray technology is a powerful genomic approach that enables researchers to quantify the expression levels of large numbers of genes simultaneously in one single experiment. Arrays can be single-channel (one-colour, cf. Affymetrix technology), which quantify the absolute expression of genes in specific experimental conditions, or two channel (two-colour, cf. cDNA technology). A key purpose of a two-colour microarray experiment is the identification of genes which are differentially expressed in two samples. Although this technology has given an enormous scientific potential in the comprehension of gene regulation processes, many sources of systematic variation can affect the measured gene expression levels. The purpose of data normalisation is to minimise the effects of experimental and/or technical variations, so that meaningful biological comparisons can be made and true biological changes can be found within one and among multiple experiments. Several approaches have been proposed and shown to be effective and beneficial in the reduction of systematic errors within and between arrays, both for single- and for double-channel technology [1-3]. Some authors proposed normalisation of the hybridisation intensities, while others preferred to normalise the intensity ratios. Some used global, linear methods, while others used local, non-linear methods. Some suggested using spike-in controls, or housekeeping genes, or invariant genes, while others preferred all the genes on the array.

In general, microarray normalisation can be divided into normalisation within arrays, for the correction of dye effects, and across arrays, for the balance of the distribution differences among experiments. Several pre-processing techniques recently proposed for two-channel technology allow the joint normalisation within and across experiments, as reported in the original papers ([4] for the vsn/glog and [5] for the q-splines). Glog and q-spline transformations, in fact, are performed on the gene expression matrix where the two channels are considered separately, allowing systematic bias reduction within and across arrays. Although several normalisation procedures have been proposed, it is still unclear which method uniformly outperforms the others under different experimental conditions. Recent works [6-8] compare, through simulated data, normalisation methods in terms of bias, variance, mean square error or leave-one-out cross-validation classification error. If we consider the two-channel technology, Park et al. [7] show that, in some cases, intensity dependent normalisation performs better than the simpler global normalisation, while [3,9] raised the concern that removal of spatial effects may add additional noise to normalised data, suggesting that a safe alternative is to remove the intensity effect only at a local level. Thus, the evaluation of normalisation's effects in microarray data analysis is still an important issue, since subsequent analyses, such as tests for differential expression, could be highly dependent on the choice of the normalisation procedure. For example, Durbin et al. [10] show that the log-transformed expression ratio has a greatly inflated variance for expression values close to 0. This effect penalises differential expression, especially for high expression levels. Hypothesis tests for differential expression may in fact be more effectively performed on data that have been transformed so as to have constant variance. Hoffman and colleagues [11] compare the effect of different normalisations on the identification of differentially expressed genes within Affymetrix technology and using a real dataset. They observe, by comparing lists of genes, that the normalisation has a profound influence on the detection of differentially expressed genes.

Moreover, the MicroArray Quality Control (MAQC) [12] project, which is specifically designed to address reproducibility of microarray technology by comparing results obtained across different array platforms, chooses the statistical analysis on the base of the normalisation and gene selection technique as the crucial steps in order to improve reproducibility [13].

When microarray experiments are adopted with diagnostic purposes, this result appears to be fundamental because scientists are looking for a list of a few pathology marker genes. Marker genes can be defined as genes whose expression profiles are discriminating between case and control samples. It is likely to suppose that the complete list of markers of a condition is composed by hundreds of genes, highly correlated and mostly implicated in few signalling cascades. Only few of them lie upstream these signalling cascades and are responsible of the differential expression of all the others genes. Hence, if different pre-processing have an impact on the identification of differentially expressed genes, they could lead to different lists of markers. The aim of this work is to compare and evaluate the impact of various normalisation procedures proposed for two-channel array technology on the identification of marker genes. We shall use both simulated and real data derived by cDNA and oligo microarray (two-colour technique).

The use of a simulation approach allows us to study the sensitivity and specificity of the tests after normalisation and to compare different approaches' performances. However, simulation of DNA microarray data can be questioned, mainly because (i) the relation between expression and experimental factors involved is not theoretically established, and (ii) the statistical distribution of differential expression given by various causes across genes is still controversial. In order to address such issues, we adopt two different classes of simulation models.

Although we found a limited difference of sensitivities and specificities for the tests after each normalisation, the study highlights a strong impact in terms of gene ranking, resulting in different levels of agreement between competing normalisations. Finally, we show that the combination of two normalisations, such as glog and lowess, that handle different aspects of microarray data, is able to outperform the other individual techniques.

## 2 Results and Discussion

### 2.1 Simulated data

**Figure 1:**
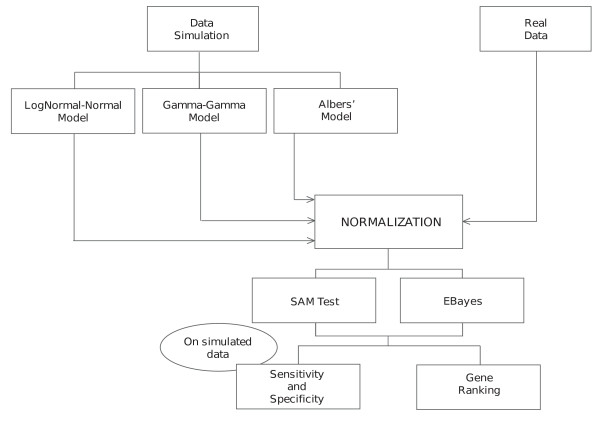
**Schema of the normalisations and analysis performed on the simulated data and real data**.

**Figure 2:**
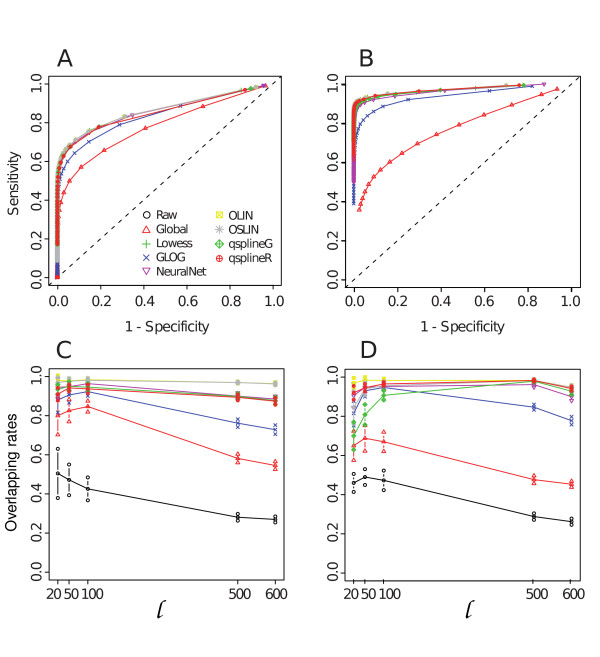
**LNN and GG models with non-linear bias**. Specificity and sensitivity (panel A and B) and average overlapping rates with 95% confidence interval of top ranking gene lists detected as differentially expressed between lowess and the others normalisations.

Figure 1 summarises our approach and Additional file 1 shows an example of MA plots obtained by our simulation models. Additional file 3 and Figure 2 pictures results from the GG and LNN simulation models (see Section 4.2.1); Additional file 2, Figure 3 and Figure 4 refer to Albers' model (see Section 4.2.2), with different levels of background (panel A and B: 10%, panel C and D: 50%, panel E and F: 150%). As this last simulation model can produce negative expression values, we investigate results for both the cases in which negative values are replaced (panels B, D, F of relevant figures) and kept as such (panels A, C, E). This is because it is a common – but incorrect – practice in microarray analysis to replace negative values with arbitrarily small positive values, so that normalisations based on log expression ratios can still be employed.

**Figure 3:**
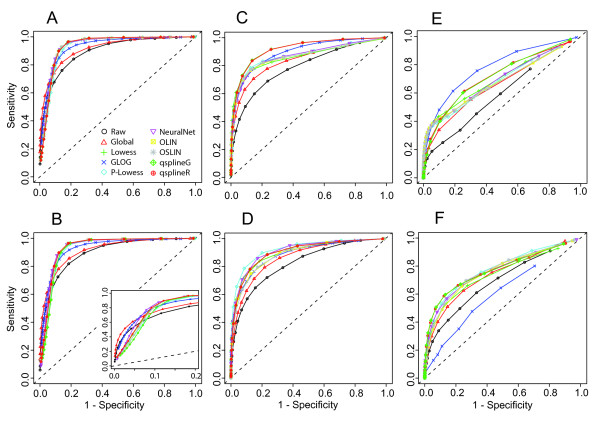
**Specificity and sensitivity curves for Albers' model with increasing percentage of background level with respect to expression level with and without replacing negative values**. Panel A, C, E with 10%, 50% and 150%, respectively, background levels without negative values replacement; panel B, D and F with 10%, 50% and 150%, respectively, background levels with negative values replacement. See Simulation Plan Section for normalisation codes.

**Figure 4:**
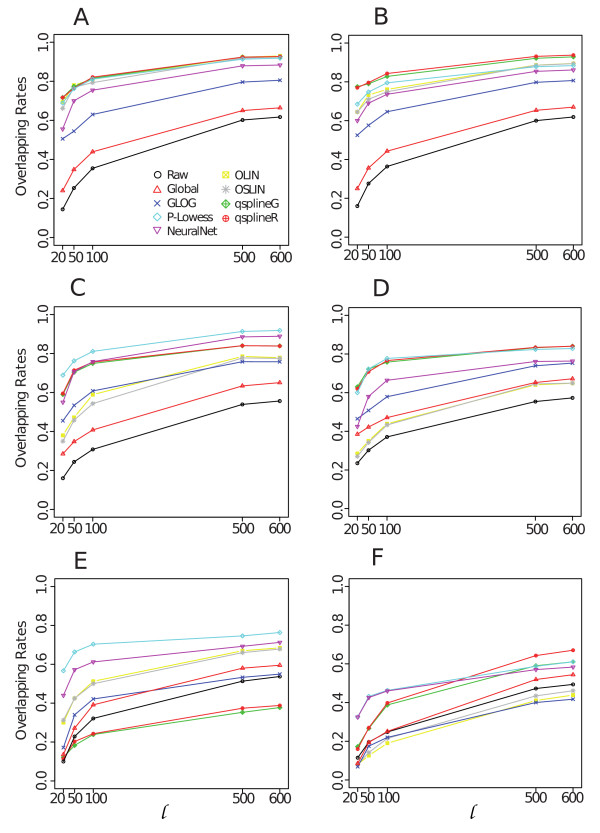
**Overlapping rates of top ranking gene lists detected as differentially expressed between lowess and the others normalisations**. Panel A, C, E: results obtained from data generated by Albers' model with 10%, 50% and 150%, respectively, background levels without negative values replacement; panel B, D, F: results obtained from Albers' model with 10%, 50% and 150%, respectively, background levels with negative values replacement.

To ease reading of the results, we performed the comparisons in two stages, involving: 1) q-splines, quantile, enhanced quantile and enhanced q-spline, and 2) the best normalisation obtained at step 1) with all the other techniques.

We found that quantile and q-splines equally perform in terms of specificity and sensitivity across all the simulated scenarios both for GG/LNN models [data not shown] and for Albers' model [Additional file 2]. On the contrary, surprisingly, enhanced quantile and enhanced q-spline show extremely reduced performances [Additional file 2]. We deeply investigated this result. We found that, in all the experimental scenarios, the additional steps performed by the enhanced method after the quantile and q-spline normalisation do not recover further relevant information from the residual matrix (see Methods for more details). Then, both with SAM and with EBayes test we observed a strongly reduced FDR estimate that increases the number of false positives genes and strongly reduces the test sensitivity. In the light of these results, we decided to proceed by taking into consideration in step 2 only q-splines normalisations. In the following, step 2 results are reported.

In case of GG and LNN models without systematic bias (Additional file 3 panels A, B) and of Albers' with 10% of background level (Figure 3A, C), all the normalisations show a similar performance. Global and glog normalisations seem to perform slightly worse than the others, but using empirical confidence interval this difference is not significant (data not shown). However, when a systematic bias is included (Figure 2, panels A and B) and with increasing background levels (Figure 3, panels C and E), the normalisations respond differently. In particular, if we consider Albers' model, q-spline and glog seem to increase performance showing the best level of specificity and sensitivity. This result can be explained by the presence of negative values. Negative values in normalisations based on log-ratio intensity transformation are necessarily treated as missing (log transformation is not defined for negative values). The replacement of negative values with an arbitrarily known small value (Figure 3B, D and 3F) has a general effect of slightly reducing specificity and sensitivity after all normalisations. The major effect is evident on the glog transformation that shows a dramatically reduced sensitivity in case of 150% background level (Figure 3 panel F).

Differences among sensitivity and specificity of the normalisations in Albers' simulated matrices have been quantified through the area under the ROC curves (AUC). Normalisations are ranked according to their AUC so that the bigger the rank, the better the normalisation (Table 1). To evaluate the reproducibility of our results and the influence that the test statistic SAM had on the normalisations comparison, we re-calculated and compared ROC curves using a different test statistic. ROC curves and the ranking of normalisation obtained through AUC using EBayes test are reported in Additional file 4 (panels A-F) and Additional file 5, respectively. It is worth noting that results obtained with the EBayes test are totally in agreement with those obtained with SAM test.

**Table 1:** Area Under the Curve (AUC) of specificity and sensitivity of SAM test after the normalisations, for Albers' model with increasing percentage of background level with and without replacing negative values.

	10% bg		10% bg replaced		50% bg		50% bg replaced		150% bg		150% bg replaced	
normalization	AUC	rank	AUC	rank	AUC	rank	AUC	rank	AUC	rank	AUC	rank

Raw	0.9	1	0.9	1	0.79	1	0.82	1	0.55	1	0.68	2
Global	0.91	2	0.91	2	0.84	2	0.86	2	0.66	2	0.74	4
GLOG	0.93	3	0.93	3	0.9	8	0.89	3	0.78	10	0.58	1
Lowess	0.94	6	0.93	3	0.86	3	0.9	6	0.69	6	0.73	3
P-Lowess	0.94	6	0.93	3	0.87	4	0.91	9	0.68	5	0.78	10
NeuralNet	0.93	3	0.93	3	0.87	4	0.91	9	0.69	6	0.76	5
OLIN	0.94	6	0.93	3	0.87	4	0.89	3	0.67	3	0.76	5
OSLIN	0.94	6	0.93	3	0.87	4	0.89	3	0.67	3	0.77	7
qsplineR	N0.94	6	0.93	3	0.92	9	0.9	6	0.72	8	0.77	7
qsplineG	0.93	3	0.93	3	0.92	9	0.9	6	0.72	8	0.77	7

For each simulated scenario is also reported the ranking of the normalisations according to the AUC: the bigger the rank, the better the normalisation.

Differences observed in normalisations performance is evidently reflected in the gene ranking (Figure 2C, D and Figure 4). We find that, on the first 100 genes, the highest overlapping rate with the lowess list is around 80% with values going down to 30% (Figure 4A, C and 4E, and Additional file 6A, C and 6E). Agreement tends to reduce even more when comparing gene lists with replaced negative values (Figure 4 panel B, D and F and Additional file 6B, D and 6F). These results have been confirmed using EBayes test [Additional file 4 panels G-L].

In general, we observe that the OSLIN procedure is essentially equivalent to OLIN, suggesting that the further scaling factor introduced in OSLIN is redundant.

Lowess and OLIN tend to show similar performances, which implies that the optimal estimate for the smoothness parameter is usually close to the default one. However, in case of the well-known MA-plot "arrow head effect", typical of an array characterised by a large proportion of small constant values [Additional file 1 panel F] the optimisation procedure erroneously captures an arrow head effect trend, while lowess with smoothing parameter set to the default value (0.4) ignores this trend.

**Figure 5:**
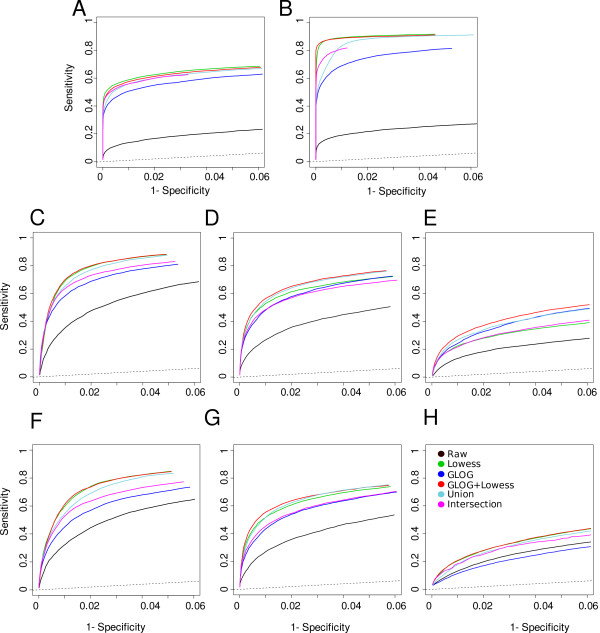
**Specificity and sensitivity obtained after glog, lowess, combination of glog and lowess, union and intersection of lists of genes obtained by glog and lowess performed separately**. Panel A and B: GG and LNN models, respectively. Panel C, D, E: Albers' model with 10%, 50% and 150%, respectively, background levels without negative values replacement; panel F, G, H: Albers' model with 10%, 50% and 150%, respectively, background levels with negative values replacement.

The highly similar level of sensitivity and specificity of most normalisations, jointly with a poor overlapping in the gene lists, suggests that different pre-processing methods could be able of capturing alternative aspects of microarray data, for example by identifying complementary lists of marker genes. Even if the identification of the best normalisation procedure seems to be unfeasible, the combination of different procedures could represent an efficient alternative. Then, we evaluate the performance in terms of specificity and sensitivity on simulated datasets of glog and lowess normalisations in the following scenarios: (i) the union of the gene lists obtained separately from glog and lowess normalisations, (ii) the intersection of the gene lists obtained separately from glog and lowess normalisations, and (iii) the list obtained from the combination of glog and lowess normalisations. This last scenario should avoid missing negative values in case of a high level of background and guarantee an efficient intensity dependent normalisation. In addition, lowess normalisation effectively removes biases within each slide but does not account for differences across multiple slides, which, on the contrary, are provided by glog. Specificity and sensitivity have been calculated by varying the number of top ranking genes from 10 to 600 with step 20. In case of scenarios (i) and (ii), the number of top ranking genes in both lists have been selected in order to obtain a union and intersection list of desired size. Figure 5 shows the results either for GG, LNN or Albers' models. In simulated datasets, the combination of glog and lowess proves to be better than the other combinations. This result suggests that combining these two normalisations is advantageous in terms of identification of differentially expressed genes.

### 2.2 Real data

Microarray normalisations are based on at least two fundamental assumptions: i) only a small portion of spots is differentially expressed and ii) differentially expressed spots are homogeneously distributed among the over and the under expressed ones. These assumptions are reasonable for most of large-scale genome experiments where only a small proportion of the entire genome is involved in the biological process studied, but could fail in case of a platform with only limited genome coverage or for specific experimental treatments.

Therefore, we selected real datasets in order to consider different experimental situations. We chose experiments obtained with two spotted cDNA and two spotted oligos platforms and characterised by i) a weak response in terms of differential expression, ii) a strong response in terms of differential expression, iii) a large number of negative values replaced, iv) a large number of negative values kept as such. Table 2 briefly describes the datasets' characteristics.

**Table 2:** Description of real datasets used for the analyses.

	Dataset	Organism	Platform	Features	Expected DEG	Negative spots	Replaced
A	Baird et al.	Homo sapiens	cDNA	12,600	2,045 (0.16)	7,444 (0.59)	With zero
B	Urban et al.	S. Cerevisiae	Oligo	10,789	3,719 (0.34)	None	-
C	Smith et al.	S. Cerevisiae	Oligo	25,240	27 (0.004)	524 (0.08)	No
D	De Pittà et al.	Homo sapiens	cDNA	9,984	1,353 (0.13)	None	-

For each dataset the table reports the organism, the platform, the number of features, the number of expected differentially expressed genes (DEG) (in brackets the expected proportion), the mean number of negative spots per array (in brackets the mean proportion), and if the negative spots are replaced or not. Note that in dataset C each array contains four replicates of each gene, giving a total of 6,130 unique features. Therefore, the proportion of genes expected to be differentially expressed has been calculated on the total of unique features. Expected number of DEG is calculated as the average number of differentially expressed genes obtained with SAM test after all the normalisations used in the study.

**Figure 6:**
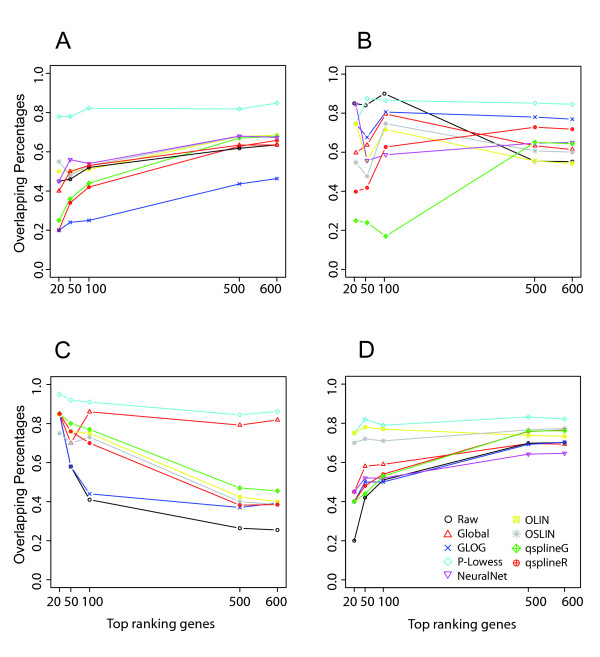
**Overlapping rates of top ranking gene lists detected as differentially expressed between lowess and the others normalisations in dataset A (panel A), dataset B (panel B), dataset C (panel C) and dataset D (panel D)**.

Figure 6 shows the overlapping rates between normalisations for datasets A-D. In general, these results show a general agreement with those obtained on simulated data. Dataset B is characterised by the absence of negative values and a strong differential expression (rapamycin treatment on *Saccharomyces cerevisiae*) symmetrical among up and down regulation. The mean overlapping percentage is about 70%. We note that normalisations on dataset B lead to lists of genes with a higher overlapping rate with respect to the others. On the other hand, datasets C and A are characterised by the presence of negative spots and show the worst overlapping percentage between glog and lowess. Through our simulation results we are able to differentiate both situations. Replacement of negative values (as in dataset A) has a negative effect on glog normalisation, dramatically decreasing specificity and sensitivity of SAM test (Figure 3F), while the presence of negative values kept as such (as in dataset C) negatively affects lowess-type normalisations (Figure 3E). Thus, differences among glog and lowess reflect i) the failure of glog to effectively normalise dataset A and ii) the ability of glog to outperform the other normalisations in dataset C. The average overlapping of dataset D is slightly less then 60%.

According to our results, differences among normalisation performances seem to be independent from array platforms and from the type of differential expression response (under the condition that deregulation is symmetrically distributed among over and under expression) but rather are dependent from the presence and the way of dealing with negative values.

Performance of the combination of glog and lowess, as well as of lowess and enhanced quantile, have been carried out through the identification in dataset D of a small list of true positives, retrieved from published biomedical research literature by Bioinformatics Organization Inc. Here, we include the combination of lowess and enhanced quantile in order to evalute if the performance of enhanced normalisation (poor in simulated data, see Additional file 2) could be badly influenced by the Workman et al. [5] strategy used for two-channel technology. The use of lowess and, then of enhanced quantile (on normalised log ratio), should avoid this possibility. Table 3 shows the rank of true positive features obtained after glog, lowess, union and intersection of gene lists obtained from glog and lowess separately, the combination of glog and lowess and the combination of lowess and enhanced quantile. The smaller the rank of true positives, the more efficient is the normalisation. The combination of glog and lowess shows the smallest rank for most of the genes, suggesting a better performance compared to the others and confirming, even with real data, the poor performance of the combination of lowess and enahnced quantile.

**Table 3:** Test statistic ranks of the 10 true positive genes obtained after glog, lowess, combination of glog and lowess, combination of lowess and enhanced quantile, union and intersection gene lists.

ID	Symbol	lower	FC	upper	raw	glog	lowess	glog+lowess	lowess+enhancedQ
BL-003F10	PBX2	-2.02	-3.42	-4.83	130	474	158	**26**	179
BL-003F10	PBX2	-1.95	-3.36	-4.77	139	486	172	**32**	178
2-014C09	BTG1	-0.09	-1.94	-3.8	1080	7088	3302	741	**556**
BL-010C07	DLEU2	-0.99	-1.91	-2.83	275	902	529	**163**	613
BL-010C07	DLEU2	-0.81	-1.79	-2.77	375	955	538	**184**	624
2-025D04	CEBPA	2.51	1.53	0.54	527	1976	**461**	1462	2191
2-029B10	FUS	-0.2	-1.51	-2.81	746	7268	**543**	855	1101
2-025D04	CEBPA	2.52	1.31	0.11	**660**	5228	1243	1678	677
2-029B10	FUS	-0.41	-1.23	-2.05	685	1236	820	**385**	1421
2-019A01	CAV1	-0.05	-1.2	-2.34	1960	6519	1962	8034	**1595**

FC: fold change; upper and lower: confidence interval upper and lower bounds; Symbol: gene symbol.

## 3 Conclusion

The main aim of this research effort was to report on an exploratory study for benchmarking the impact of several normalisation techniques in detecting differentially expressed genes. The documentation of the simulation models, the experimental setup, the analysis on real data should enable the reader to assess the robustness and scope of the benchmarking.

Results were presented in terms of mean sensitivities and specificities and mean overlapping rates of gene ranking lists. Summarising our results, we are able to say that, in general, the comparison of the sensitivities and specificities shows limited difference in impact of preprocessing over the range of operating conditions. On the other side, the study highlights an evident impact in terms of gene ranking agreement. With equal levels of specificity and sensitivity, gene lists differ from an average of 40% of the genes with Albers' model to an average of 20% with GG and LNN models (with bias included).

This might have important effects on some microarray-based research, where, through gene ranking and discriminant analysis, a small set of genes is selected to become markers of the studied pathology. Our study suggests that, putative marker genes obtained with different normalisations could be substantially different.

This is more evident in case of replacement of null or negative values where the higher the number of replacements, the lower the sensitivity and specificity of the test, and the lower the rate of agreement of gene ranking. Therefore, the best pre-processing action might depend upon the distribution of the data, and a careful exploratory analysis is called for before applying normalisation.

Real datasets (selected in order to cover different experimental conditions) confirmed these results. Differences among normalisation performances seem to be independent from array platforms and from the type of differential expression response (under the condition that deregulation is symmetrically distributed among over and under expression), but seem to depend on the presence and the handling of negative values. We also show that the combination of glog and lowess may avoid the drawbacks given by the negative values (due to highly level of background) and may guarantee an efficient intensity dependent normalisation. The advantage of the combination is much more evident without replacement of negative values.

## 4 Methods

### 4.1 Normalisation essentials

In this section, we give an overview of the essentials of the normalisation techniques that are taken up in our comparative study. Since we are unable to cover all of the technical details in this article, we refer the reader to the relevant literature.

#### Global normalisation

Global normalisation [1] is usually directed to balance the different incorporation effciencies of the two fluorophores (Cy3 dye and Cy5 dye) in the two-channel technology. Global intensity normalisation relies on the assumption that the quantity of mRNA is the same for both labelled samples. Furthermore, assuming a symmetrical distribution of over- and under-expressed genes for thousands of genes in the array, these changes should balance out so that the total quantity of RNA hybridising to the array from each sample is the same. Consequently, the total integrated intensity for all spots should be the same in both the Cy3 and Cy5 dyes. Under this assumption, a normalisation factor can be calculated and used to re-scale the intensity for each gene in the array.

#### Lowess normalisation and its variants

Lowess normalisation [14] relies on the use of a non-linear regression technique (the widely used LOWESS, LOcally WEighted Scatterplot Smoothing) based on robust local regression of the log ratios of Cy3/Cy5 on overall spot intensity Cy3*Cy5 (the LOWESS smoother for the so called MA-plots, where M is the log transformation of Cy3/Cy5 and A is the log transformation of the squared root of Cy3*Cy5). The normalised M can be written as

*M' *= *M *- *c*(*A*),

where *c*(·) is the LOWESS smoother. The normalisation model is based on the assumption that a significant fraction of the probes in the array is expressed at similar levels.

Print-tip lowess normalisation (P-lowess hereafter) proposed by Yang et al. [14] takes into account possible spatial intensity artifacts introduced by robot print-tips during the spotting step. P-lowess is based on individual linear local regression (lowess) limited to a single print-tip group. In this way, each print-tip group has its own normalisation curve. The formula for the normalisation is

*M' *= *M *- *c*_*i*_(*A*),

where *i *= 1, ..., *k *is the *i*-th print-tip group.

Futschik and Crompton [15] show that the arbitrary use of local regression parameters can severely compromise the quality of normalised data. Parameter choice has commonly been left to the user, and instructions on how to adjust the parameters to the underlying data structure are generally not given. In order to overcome these limitations, Futschik and Crompton [16] introduce two normalisation schemes, Optimized Local Intensity-dependent normalisation (OLIN) and Optimized Scaled Local Intensity dependent normalisation (OSLIN), based on iterative local regression and model selection. OLIN is based on iterative local regression where parameters are optimised in each regression step by generalised cross-validation. OSLIN comprises OLIN procedure with a subsequent optimised scaling of the range of log-intensity ratios across the spatial array dimensions.

#### Neural Networks

Tarca and colleagues [17] propose a method based on a robust neural network model that uses the log-intensity ratio (M) as the independent variable, and the average log-intensity (A) as well as spatial location of spots as predictors. Resistance to outliers is provided by assigning weights to each spot based on how distant their M values are from the median over the spots whose A values are similar, and also by using pseudo-spatial coordinates instead of spot row and column indices. The authors use a simple feed-forward neural network with sigmoid activation function suggesting three neurons in the hidden layer.

#### q-splines

Q-spline normalisation [5] uses the quantiles from each array and the target to fit a system of cubic splines to normalise the data. The target should be the (geometric) mean or median of each probe. The authors propose splines for their robustness in representing almost any smooth relationship, including the linear one. Using quantile information provides a much easier fitting problem and avoids fitting the pairwise data directly, which often requires robust regression techniques.

#### Quantile

Originally, quantile normalisation [6] was proposed as an across arrays normalisation suitable for single channel technology. We decided to evaluate the quantile performance using the same strategy proposed by Workman et al. [5] and Wu et al. [8] for the q-splines. The goal of quantile normalisation is to give the same empirical distribution of a target reference to each array. Following Wu et al. [8], the reference target is defined as the geometric average of Cy3 (or Cy5) channel. Considering the simple case of dimension *n *= 2, if two data vectors have the same distribution, a quantile-quantile plot will have a straight diagonal line, with slope 1 and intercept 0. Thus, if the quantiles of two data vectors are plotted against each other and each of these points are projected onto the 45-degree diagonal line, we obtained a transformation that gives the same distribution to both data vectors. Quantile normalisation is the generalisation to *n *dimensions of the above transformation.

#### Enhanced procedure

The enhanced normalisation procedure recently proposed by Hu and He [18] uses singular value decomposition (SVDs) of the normalised microarray data matrix and of the correspondent residual matrix (defined as the difference between the original matrix and the normalised one) to allow users to filter out noise and recover relevant information that might be lost in a given normalisation procedure. The goal of the procedure is retaining maximal relevant information in gene expression profiles. For an exhaustive description of the methodology, see the original paper by Hu and He [18]. In this study, we apply the enhanced procedure to quantile and to q-spline normalisations.

#### Variance stabilising normalisation

As alternative to any other pre-processing technique, Rocke and Durbin [19] and Huber and colleagues [4] present independently a family of variance stabilising transformations based on the generalised logarithmic transformation (glog).

Glog assumes that raw gene expression intensities, *y*, can be modeled as the sum of three components: (i) average background noise, *α*, (ii) true expression level, *μ*, multiplied by an exponential error term, *η*, normally distributed with zero mean and variance $\sigma_{\eta }^{2}$, and (iii) an additive error term, *ε*, normally distributed with zero mean and variance $\sigma_{\varepsilon }^{2}$, as

*y *= *α *+ *μe*^*η *^+ *ε*.

Glog transformation, which can be equivalently applied to single- and double-channel microarray technology, should achieve absence of relation between mean and variance of the expression. It can be written as

$$glog(y)=\log\left(y-\alpha +\sqrt{{\left(y-\alpha \right)}^{2}+c}\right),$$

with $c=\frac{\sigma_{\varepsilon }^{2}}{e^{\sigma_{\eta }^{2}}(e^{\sigma_{\eta }^{2}}-1)}$

### 4.2 Simulation models

In this section, we document the simulation models that we have used in our analyses.

#### 4.2.1 Hierarchical models

##### Generation of signal intensities

We adopt a mixture model strategy as in [20]. Genes come from two different groups: differentially expressed (DE) and equally expressed (EE). Each group is modelled by its own distribution. The data as a whole are modelled by a weighted mixture of these distributions, where the weights *p *and (1 - *p*) correspond to the prior probabilities of being differentially expressed and equally expressed, respectively. If we write the expression value of the gene *g *as $y_{g_{k}}$, *g *= 1, ..., *n*, in the channel *k*, *k *= 1, 2, we have

$$f(y_{g_{1}},y_{g_{2}})=pf_{1}(y_{g_{1}},y_{g_{2}})+(1-p)f_{0}(y_{g_{1}},y_{g_{2}}).$$

According to the empirical Bayesian approach, we suppose that the intensity values of the two channels $y_{g_{k}}$ are random samples from the distribution *f*_*obs *_($y_{g_{k}}$|*μ*_*g*_) with *k *= 1, 2, respectively. In the EE case, we assume that the 2*n *values are independent, identically distributed, according to the distribution of *f*_*obs*_. Hence, under the EE hypothesis, the marginal distribution is

$$f_{0}(y_{g_{1}},y_{g_{2}})=\int \left(\prod_{i=1}^{n}f_{obs}(y_{g_{1},i}|\mu_{g})\right)\left(\prod_{i=1}^{n}f_{obs}(y_{g_{2},i}|\mu_{g})\right)\pi (\mu_{g})d\mu_{g},$$

where *π*(*μ*_*g*_) is the prior distribution of the mean signal *μ*_*g*_, representing variations in the mean intensity value of genes in the experiment [20].

Under the DE hypothesis, the latent mean $\mu_{g_{k}}$ of the sample of the channel *k *is different in each *k*. In particular, the two values of $\mu_{g_{k}}$ are drawn independently from the distribution *π*(*μ*_*g*_), leading to

$$f_{1}(y_{g_{1}},y_{g_{2}})=f_{0}(y_{g_{1}})f_{0}(y_{g_{2}}),$$

where

$$\begin{array}{l} f_{0}(y_{g_{1}})=\int \left(\prod_{i=1}^{n}f_{obs}(y_{g_{1},i}|\mu_{g})\right)\pi (\mu_{g})d\mu_{g},\\ f_{0}(y_{g_{2}})=\int \left(\prod_{i=1}^{n}f_{obs}(y_{g_{2},i}|\mu_{g})\right)\pi (\mu_{g})d\mu_{g}. \end{array}$$

We considered the two mixture models of Kendziorski et al. [20]. In the first model, named Gamma-Gamma (GG), the intensities for the replicates in both conditions (Cy3 and Cy5) are assumed to be independently generated from Gamma distributions with a constant shape parameter *α *and gene-specific random scales *λ*_*g*_, assumed to have a Gamma distribution with shape hyperparameter *α*_0 _and scale hyperparameter *ν*. In the second model, named lognormal-normal (LNN), the log intensities are assumed to be normally distributed, with constant variance *σ*^2 ^and gene-specific random means *μ*_*g*_, that are themselves normally distributed with hyperparameters *μ*_0 _and *τ*. If a gene is selected to be equally expressed, then a value for the random parameter is sampled from its prior distribution, determining the distribution from which independent replicates for both conditions are produced. If a gene is selected to be differentially expressed, then two values for the random parameter are sampled from its prior distribution, determining the two distributions from which independent replicates in each condition are produced.

##### Non-linear systematic bias

The hierarchical models GG and LNN simulate datasets without intensity dependent systematic bias. Therefore, any normalisation becomes redundant. For this reason, we decided to introduce a systematic bias effect obtained through the addition (to the log-ratio simulated by GG and LNN models) of an opportunely scaled component, inversely proportional to A.

#### 4.2.2 Albers' additive model

Differently from the previous models, Albers et al. [21] propose a model specifically drawn to include several layers of bias representative of possible experimental factors influencing microarray experiments. Albers' model has 29 parameters, 6 of which are known constants, while the others should be set by the user. The final log-expression signals, $y_{g_{k}}$, *g *= 1, ..., *n*, *k *= 1, 2, where *n *is the number of genes in the platform and *k *is the channel index, are composed by the following elements: (i) a gene expression value, $G_{g_{k}}$, (ii) an expression change for differentially expressed genes, $D_{g_{k}}$, (iii) a channel effect, *C*_*k*_, (iv) a spot pin effect, *S*_*g*_, (v) a raw background gradient signal, *b*_*g*_, (vi) a nonlinear effect, *f*_*nl*_, (vii) a fish-tail effect (inflating variance) due to the log transformation for small expression values, *t*_*d *_and (viii) a random error due to unknown factors, $e_{g_{k}}$. Then

$$\begin{array}{c} y_{g_{k}}=t_{d}(f_{nl}(b_{g}+z_{g_{k}})),\\ z_{g_{k}}=G_{g_{k}}+D_{g_{k}}+C_{k}+S_{g}+e_{g_{k}}, \end{array}$$

and the error term is assumed to be Gaussian.

### 4.3 Experimental setup

#### 4.3.1 Hierarchical models

We fixed parameter values for GG and LNN models by using estimates obtained on real datasets: for the GG model, we set (*α*, *α*_0_, *ν*) = (3.6, 2.4, 1761.19) and for the LNN model, we set (*μ*_0_, *σ*, *τ*) = (7.9, 0.164, 0.895). Under both models, the prior probability *p *of differential expression is set to 0.06.

#### 4.3.2 Albers' model

Additional file 7 reports the parameters setup used in Albers' model. Several of these values are proposed by the authors as estimates obtained on real datasets. Three different background levels have been used. The maximum of the background signal (%) relative to the non-background signal has been set to 10%, 50% and 150%. In this way, different microarray data scenarios are obtained, characterised by different proportions of negative expression values. In the first scenario (10%), expression values are mostly positive; in the last one (150%), a large number of negative values is observed. Albers' model allows the inclusion of several types of systematic biases (such as non-linear effect, fish-tail effect, background surface variation).

#### 4.3.3 Simulation Plan

For each model and set of parameters, we simulated 10 matrices with 10,000 genes expression levels on 15 experiments separately for the Cy5 and Cy3 channels. So, each simulated matrix consisted of 10,000 × 30 values (of which 15 values are Cy5 levels and 15 Cy3 levels). Each of the 10 matrices was pre-processed with 10 procedures, coded as: raw data, global normalisation, lowess, P-lowess, OLIN, OSLIN, neural network, q-spline with target Cy5 (called qsplineR) and q-spline with target Cy3 (called qslineG), glog. GG and LNN models do not account for print-tips platform geometry, therefore P-lowess normalisation was not considered in the comparison of performances of these models.

At the end of the pre-processing phase, we obtained 10 different matrices for each simulation. SAM analysis [22] and empirical Bayes test [23] were performed on each matrix. Figure 1 summarises the entire simulation plan.

To compute the average overlapping rates, we considered the following values for the length of the top ranking gene lists: 20, 50, 100, 500, and 600.

#### 4.3.4 Real Data

We used two cDNA expression datasets and two oligonucleotide datasets to validate our simulation results. All the datasets are publicly available at the GEO database.

Baird et al. [24] (hereafter dataset A) studied expression profiling of 181 tumors representing various classes of bone and soft tissue sarcomas. In this study, we selected only the 18 Ewing's sarcoma samples. The common reference was obtained by pooling sarcoma cell lines. Expression datasets and platform annotation are available on the NCBI GEO database with platform identification number GPL1977 and reference series GSE2553.

Urban et al. [25] (hereafter dataset B) analysed the rapamycin response in Saccharomyces cerevisiae. Global transcriptional analysis of rapamycin response was conducted on cells expressing either a wild-type or TOR-independent allele of Sch9. In our work, we considered only samples GSM185035, GSM185498, GSM185503, GSM185504, GSM185518, GSM185519. Expression datasets and platform annotation are available on the NCBI GEO database with platform identification number GPL884 and reference series GSE7660.

Smith et al. [26] (hereafter dataset C) studied the expression profiles of transcription factor deletion strains in the presence of oleate. mRNA levels in each of four deletion strains (delta_OAF1, delta_PIP2, delta_ADR1 or delta_OAF3) were compared to those in wild type cells by microarray analysis. There were two biological replicates for each experiment, and for each replicate both label orientations were analysed on arrays containing 4 replicate spots of each gene, resulting in a total of 16 replicate spots per gene. For our study we considered only the delta_ADR1 samples. Expression datasets and platform annotation are available on the NCBI GEO database with platform identification number GPL4287 and GPL4303, and reference series GSE5862.

De Pittà and colleagues [27] (hereafter dataset D) obtained expression profiling of bone marrow from paediatric patients with acute lymphoblastic leukemia (ALL) using a dedicated muscle cDNA array. Patients were clinically classified into B-cell ALL (9 samples), T-cell ALL (5 samples) and all compared to a common reference (commercial RNA, Stratagene, Europe) prepared from male fetal skeletal muscle. Expression datasets and platform annotation are available on the NCBI GEO database with platform identification number GPL2011 and reference series GSE2604.

By the analysis of four datasets, we are able to test the normalisation procedures in different situations: (i) either a large (dataset B) or a small (dataset C) proportion of genes expected to be differentially expressed; (ii) either cDNA (dataset A and D) or oligonucleotide (dataset B and C) microarrays; (iii) microarrays with a large number of negative spots, either replaced with zero (dataset A) or kept as such (dataset C). See Table 2 for a description of each dataset. Due to the large amount of negative spots per array, we considered for the dataset A only 6,154 genes (6,446 genes were filtered because of the presence of more than 80% of missing values on the total number of experiments).

From published biomedical research literature, Bioinformatics Organization Inc. retrieved a list of 70 genes experimentally known to be deregulated in acute lymphoblastic leukemia, and stored them in a public database available at . Of these 70 genes, only 36 were present in the custom array used by De Pittà et al. [27], among which 10 were found significantly deregulated. Therefore, in dataset A these 10 genes were considered as true positives, in order to evaluate the performance of some normalisations.

#### 4.3.5 Evaluation criteria

To evaluate the impact of the normalisation techniques in detecting differentially expressed genes in simulated datasets, we compare the results of a significance analysis based on SAM and empirical Bayes test statistic after various normalisations.

##### SAM test

SAM test statistic *d*_*g *_is defined as [22]:

$$d_{g}=\frac{{\bar{y}}_{g_{1}}-{\bar{y}}_{g_{2}}}{s_{g}+s_{0}},$$

where *s*_*g *_is the standard deviation and *s*_0 _is a positive costant, usually the 90th percentile of the *s*_*g *_distribution. Values and significance of the SAM statistic is obtained via a permutational approach [22] as follows.

1. Calculate the observed values

$$d_{g}=\frac{{\bar{y}}_{g_{1}}-{\bar{y}}_{g_{2}}}{s_{g}+s_{0}},$$

for any *g *= 1, ..., *p*.

2. Order the observed values

*d*_(1) _≥ *d*_(2) _≥ ... ≥ *d*_(*p*)_.

3. For any permutation *k *(with *k *= 1, ..., *K*) of data calculate

$$d_{g}(k)=\frac{{\bar{y}}_{g_{1}}^{\left(k\right)}-{\bar{y}}_{g_{2}}^{\left(k\right)}}{s_{g}+s_{0}}.$$

4. Order the values *d*_*g*_(*k*)

*d*_(1)_(*k*) ≥ *d*_(2)_(*k*) ≥ ... ≥ *d*_(*p*)_(*k*).

5. Define the mean quantity

$$d_{\left(g\right)}(E)=\frac{1}{K}\sum_{k=1}^{K}d_{\left(g\right)}(k).$$

To identify differentially expressed genes, the observed values *d*_(*g*) _and *d*_(*g*)_(*E*) are compared and a threshold Δ is defined such that the gene *g *is called differentially expressed if |*d*_*g *_- *d*_*g*_(*E*)| > Δ.

##### Empirical Bayes test

The empiricial Bayes test (EBayes hereafter) [23] is based on a moderated t-statistic with a Bayesian adjusted denominator similar to that proposed by Tusher et al. [22]. EBayes uses a hybrid Bayes approach in which gene variances are modelled by a prior distribution that is updated using the data to obtain posterior distribution. Then, an estimate is derived from the posterior distribution. This shrinks the observed variances towards the prior mean. Given a prior estimate *p *of the proportion of differentially expressed genes, the posterior probability that a gene is differentially expressed can be calculated. The B-statistic given by Limma is the log-odd of being differentially expressed versus equally expressed. We calculated the adjusted p-values with Benjamini and Hochberg [28] procedure in order to rank the genes; the lower the p-value, the more significant the result.

##### Performance evaluation

In our analyses, the significance analysis is used to construct sensitivity (true-positive rate) and specificity (1 - false-positive rate) for the test. For various thresholds (of Δ parameter for SAM and of adjusted p-value for EBayes), we identify the significant genes and compute the corresponding average sensitivity and specificity of the test.

Agreement among the impacts of different normalisations is also evaluated by looking at the ranking induced on the genes by the absolute value of both statistics. Genes are ordered according to the absolute value of each statistic from the highest (rank 1) to the lowest (rank *n*, where *n *is the total number of genes). Then, genes to which correspond rank less than or equal to 20, 50, 100, 500 and 600 are compared across normalisations. Taking as reference lowess normalisation, the mean rate of common genes (overlapping rate) in the two top ranking gene lists (the list obtained after the lowess procedure versus all the others) has been calculated for various lengths of the list.

Since on real datasets true positives are generally unknown, the procedures' agreement has been evaluated through the average overlapping rates in the top ranking gene lists. However, for dataset D a small list of true positive genes was available, therefore the ranks of the true positives were used to evaluate the performance of normalisations. Further, we note that, the smaller the rank, the more efficient is the normalisation.

All statistical analysises have been performed with the R statistical package freely available on . Packages used: Biobase, Ebarrays, marray, samr, vsn, OLIN, nnNORM, affy, limma.

## Authors' contributions

MSM and DR performed all the statistical analyses (simulation, normalisations, statistical tests) and results organisation. CR and MC conceived the study, participated in the design of the study and in the interpretation of the results and revised the manuscript. MC participated also in the coordination of the work. All authors read and approved the final manuscript.

## Supplementary Material

Additional file 1 **Figure S1.** Examples of typical MA plots obtained with LNN and GG models without systematic bias (panel A and B, respectively), with LNN and GG models with systematic bias (panel C and D, respectively) and with Albers'™ model with negative values (panel E) and with negative values replaced by positive constant (panel F). Red points represent differentially simulated expressed genes. Click here for file

Additional file 2 **Figure S2.** Specificity and sensitivity curves after quantile, qspline, quantile enhanced and qspline enhanced for Albers' model with increasing percentage of background level with respect to expression level with and without replacing negative values. Click here for file

Additional file 3 **Figure S3.** LNN and GG models without non-linear bias. Specificity and sensitivity (panel A and B) and average overlapping rates of top ranking gene lists detected as differentially expressed between lowess and the other normalisations. Click here for file

Additional file 4 **Figure S4.** Specificity and sensitivity curves and overlapping rates of top ranking gene lists for Albers' model with increasing percentage of background level with respect to expression level with and without replacing negative values, using the moderated t-test EBayes. Click here for file

Additional file 5 **Table S5. **Area Under the Curve (AUC) of specificity and sensitivity of the moderated t-test EBayes after the normalisations, for Albers' model with increasing percentage of background level with and without replacing negative values. For each simulated scenario, ranking of the normalisations according to the AUC is reported. Click here for file

Additional file 6 **Figure S5.** Overlapping rates of top ranking gene lists detected as differentially expressed between lowess and the others normalisations with 95% empirical confidence interval. Panel A, C, E: results obtained from data generated by Albers' model with 10%, 50% and 150%, respectively, background levels without negative values replacement; panel B, D, F: results obtained from Albers' model with 10%, 50% and 150%, respectively, background levels with negative values replacement. Click here for file

Additional file 7 **Table S2.** Parameters setting used in the Albers' simulation model. Click here for file
