# Supplementary figures and images for: A comparison on effects of normalisations in the detection of differentially expressed genes
Source: BMC Bioinformatics. 2009 Feb 13;10:61. doi: 10.1186/1471-2105-10-61 (PMC2680204; doi:10.1186/1471-2105-10-61)

**A**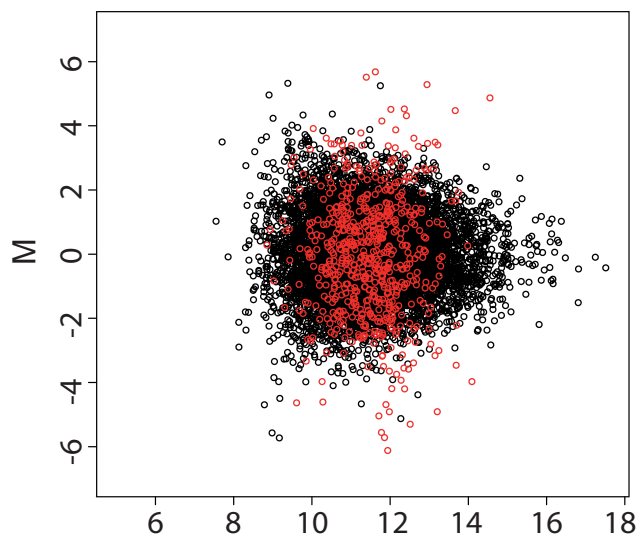**B**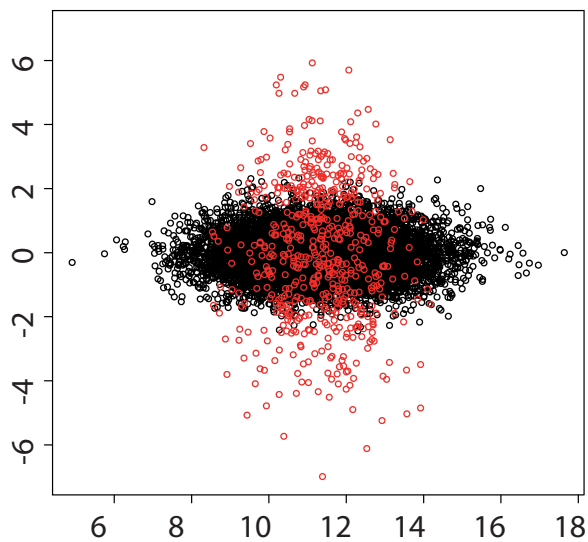**C**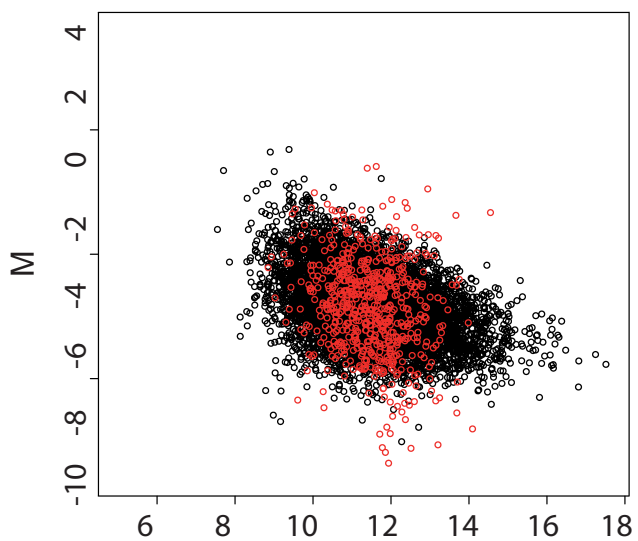**D**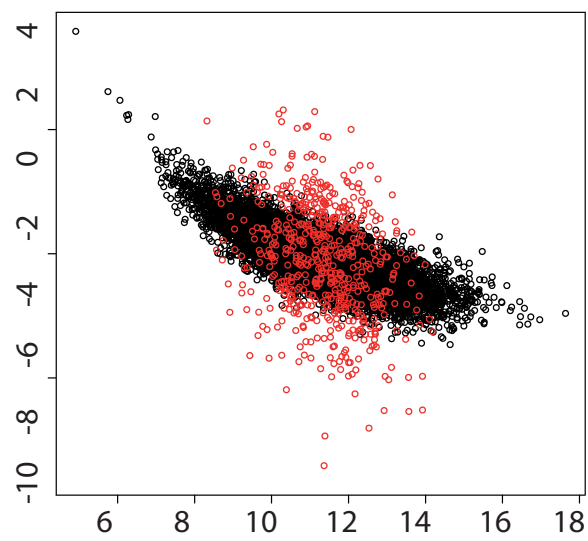**E**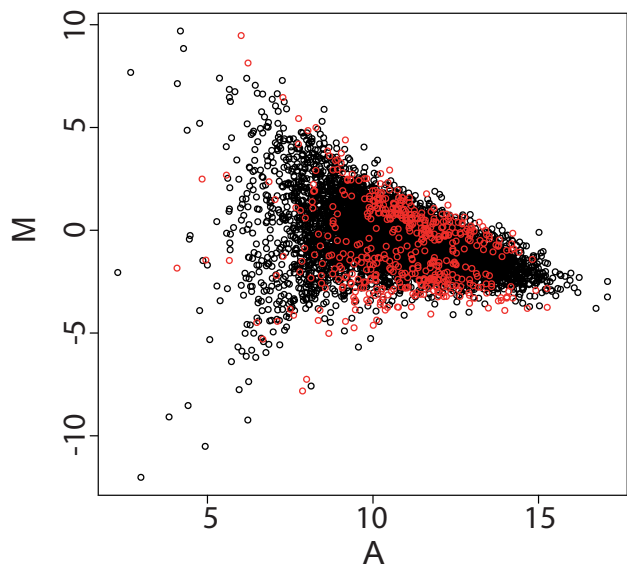**F**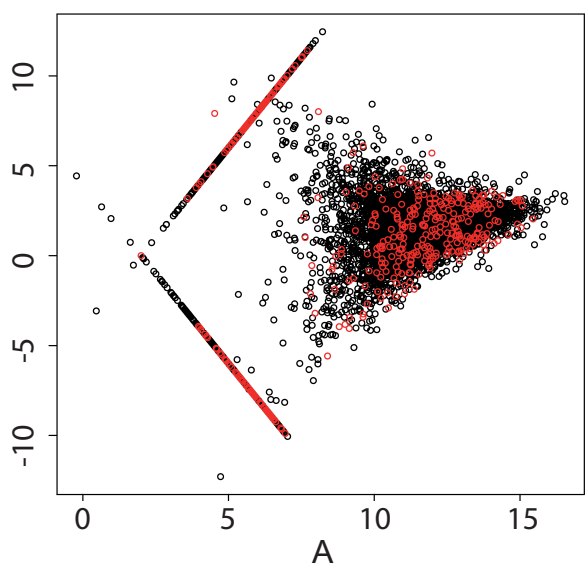

Supplement: Additional file 1 — Figure S1. Examples of typical MA plots obtained with LNN and GG models without systematic bias (panel A and B, respectively), with LNN and GG models with systematic bias (panel C and D, respectively) and with Albers'™ model with negative values (panel E) and with negative values replaced by positive constant (panel F). Red points represent differentially simulated expressed genes. [file 1471-2105-10-61-S1.pdf]

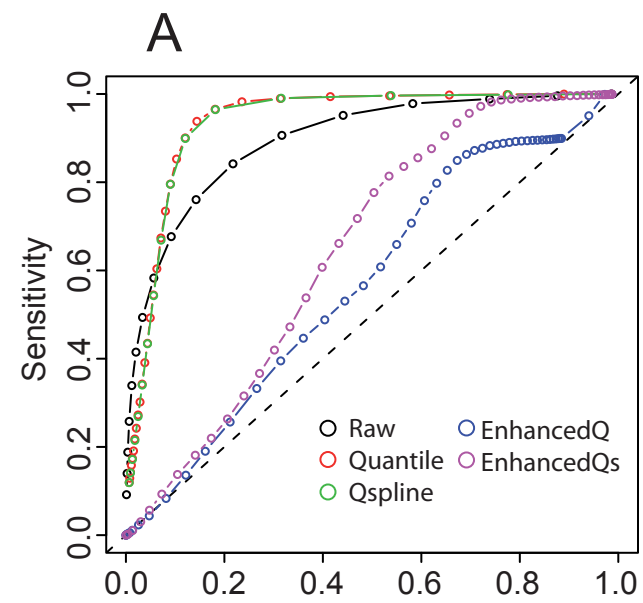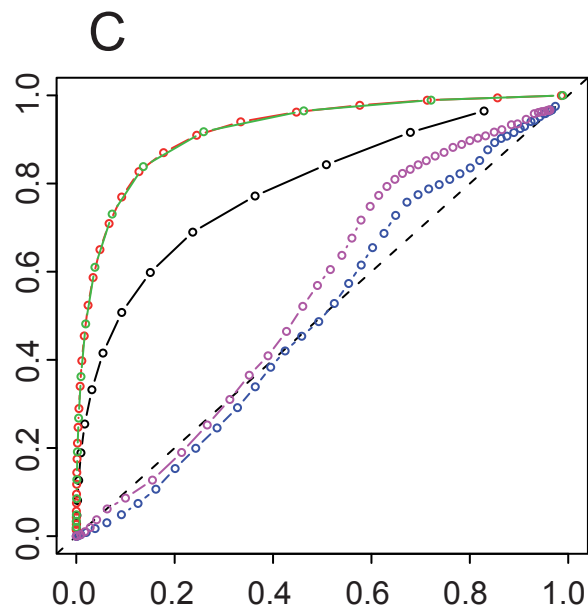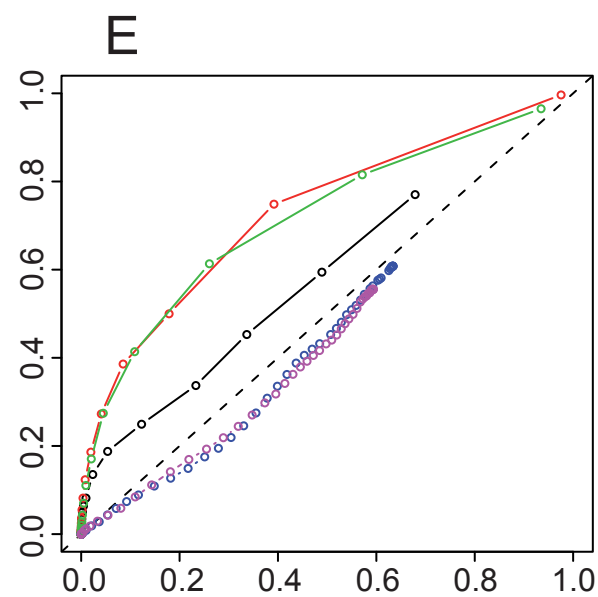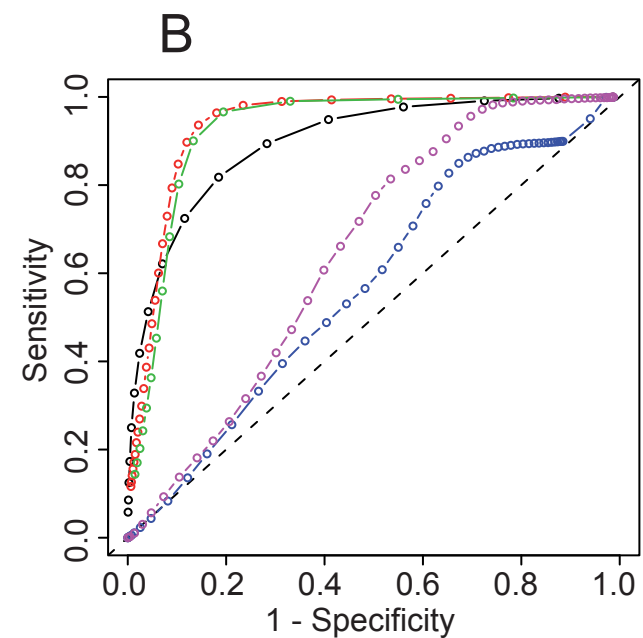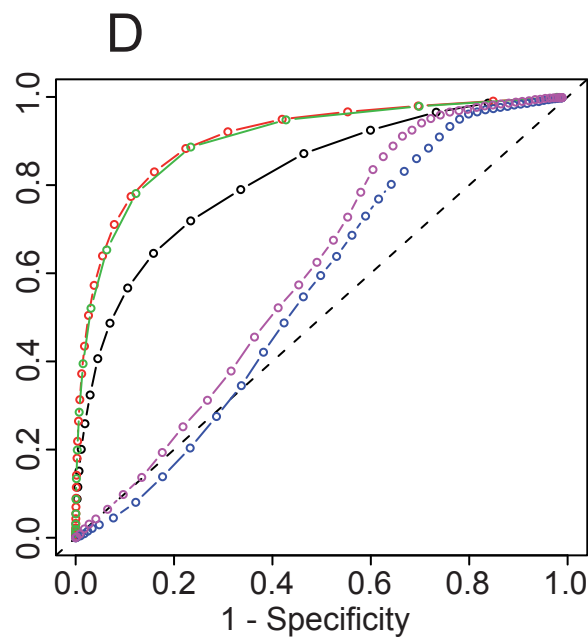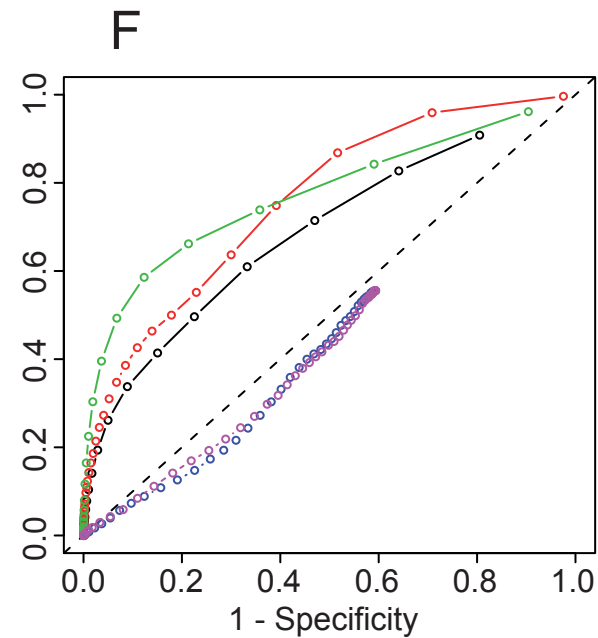

Supplement: Additional file 2 — Figure S2. Specificity and sensitivity curves after quantile, qspline, quantile enhanced and qspline enhanced for Albers' model with increasing percentage of background level with respect to expression level with and without replacing negative values. [file 1471-2105-10-61-S2.pdf]

**A**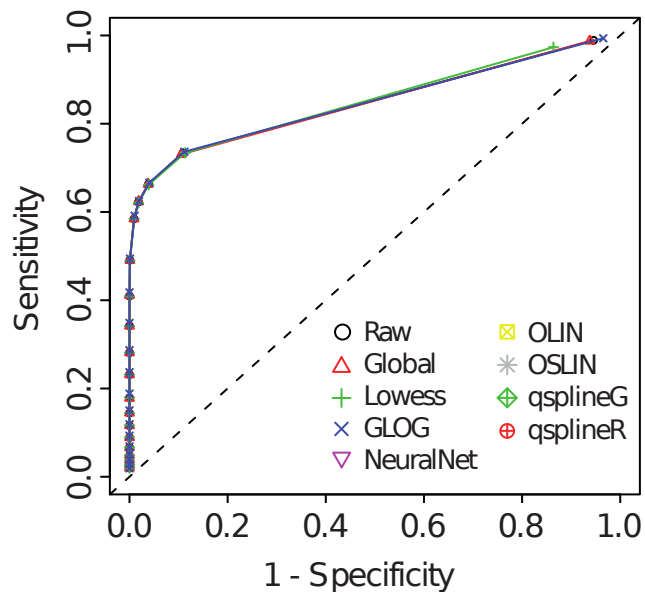**B**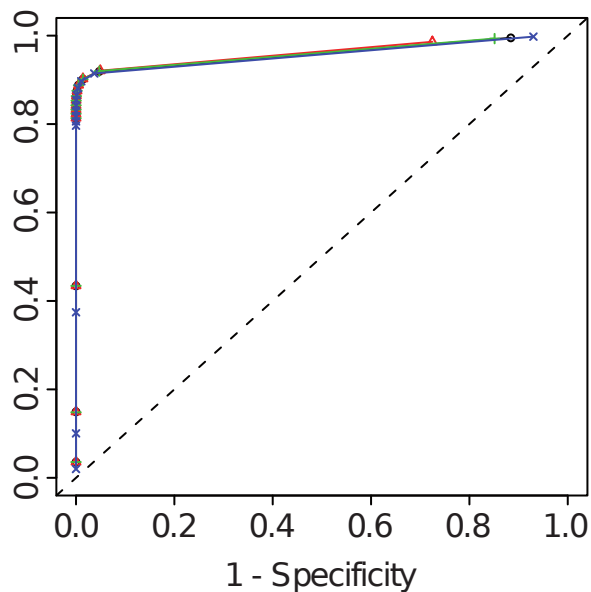**D**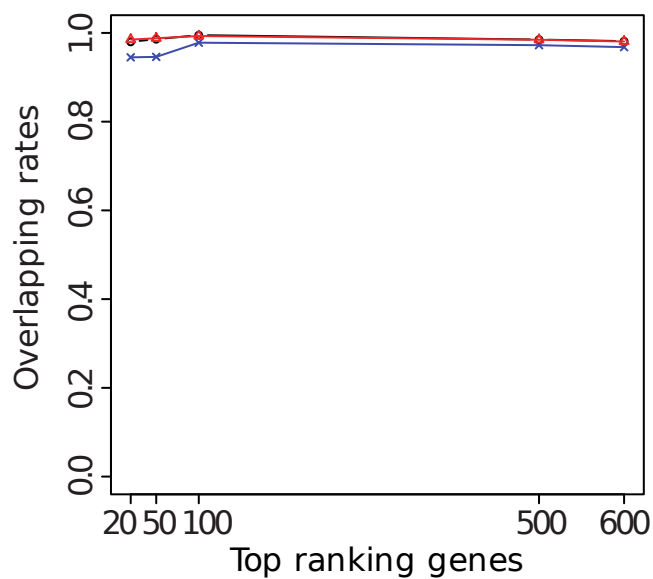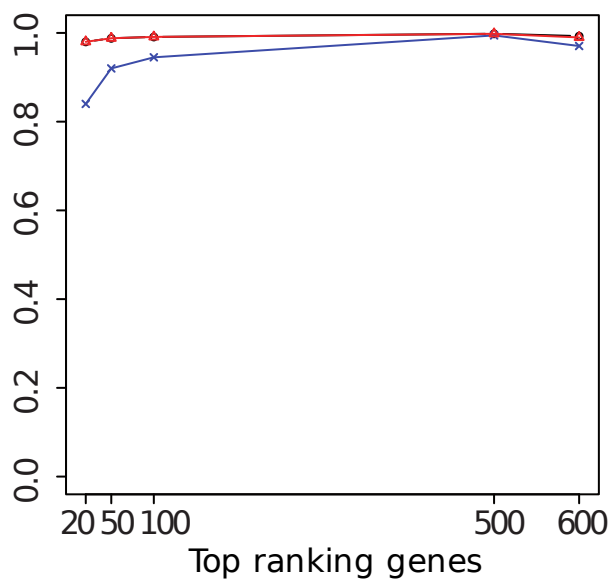

Supplement: Additional file 3 — Figure S3. LNN and GG models without non-linear bias. Specificity and sensitivity (panel A and B) and average overlapping rates of top ranking gene lists detected as differentially expressed between lowess and the other normalisations. [file 1471-2105-10-61-S3.pdf]

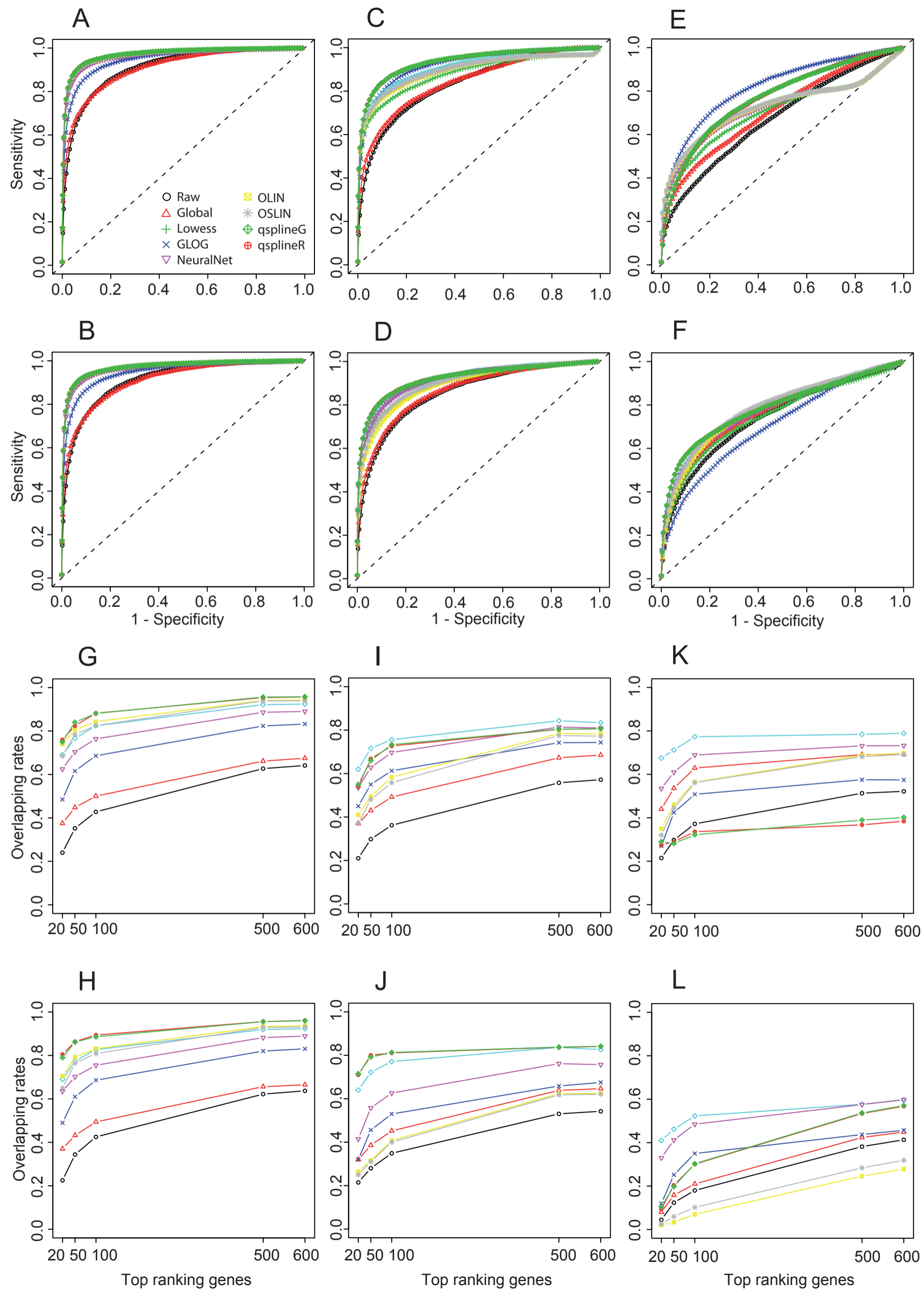

Supplement: Additional file 4 — Figure S4. Specificity and sensitivity curves and overlapping rates of top ranking gene lists for Albers' model with increasing percentage of background level with respect to expression level with and without replacing negative values, using the moderated t-test EBayes. [file 1471-2105-10-61-S4.pdf]

**A**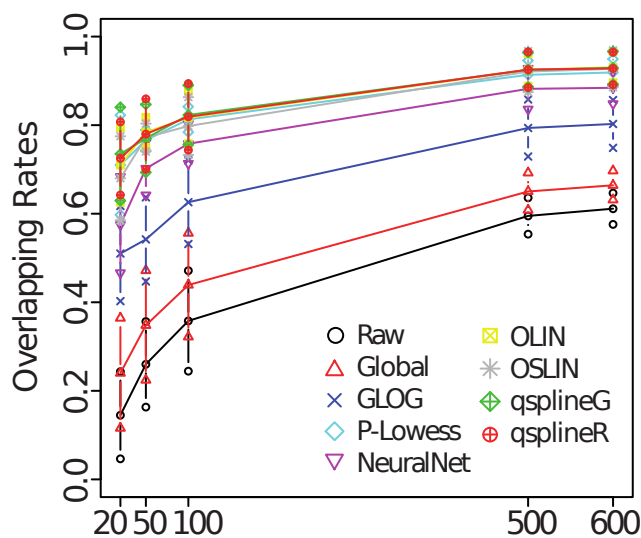**B**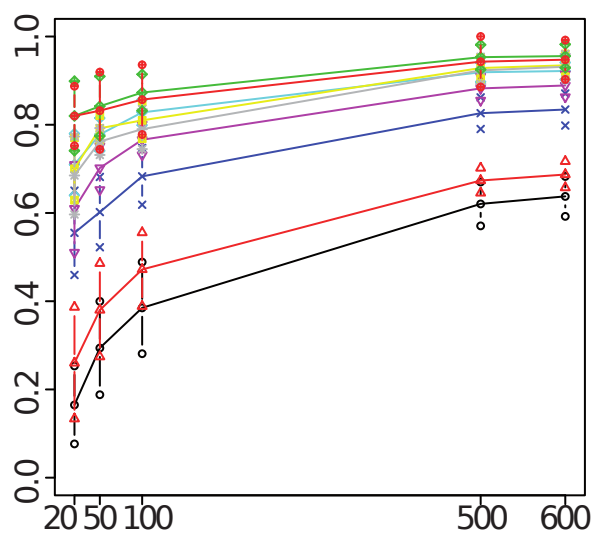**C**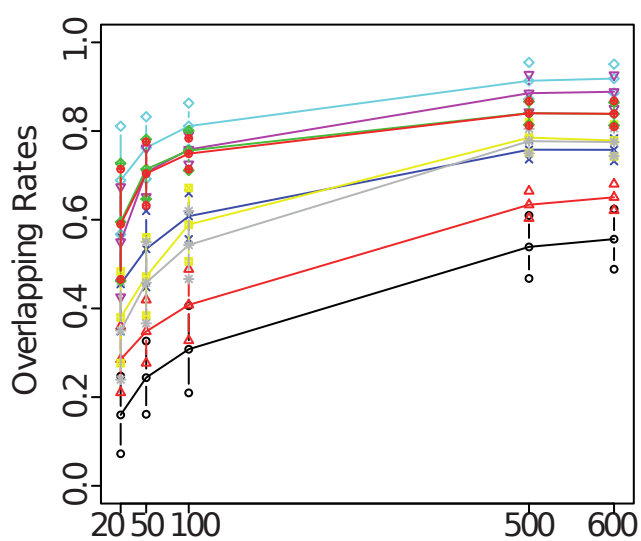**D**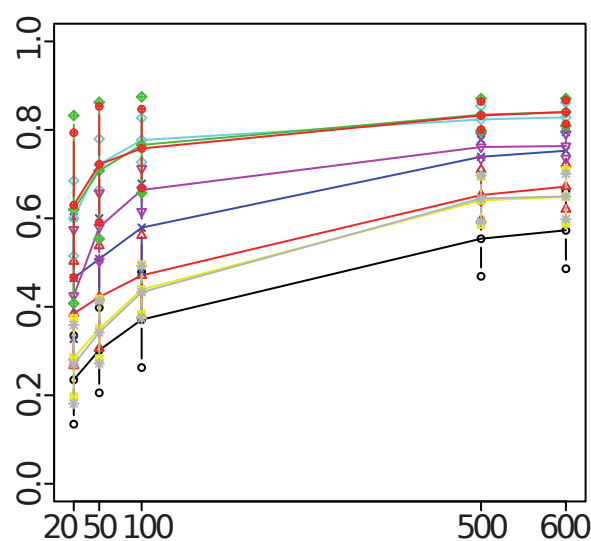**E**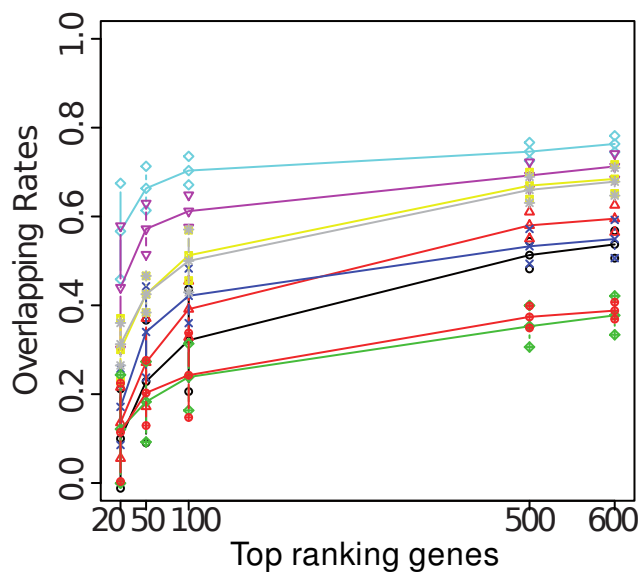**F**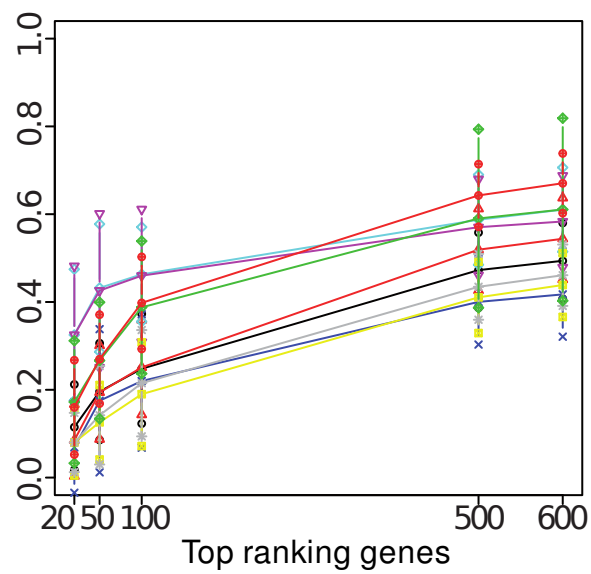

Supplement: Additional file 6 — Figure S5. Overlapping rates of top ranking gene lists detected as differentially expressed between lowess and the others normalisations with 95% empirical confidence interval. Panel A, C, E: results obtained from data generated by Albers' model with 10%, 50% and 150%, respectively, background levels without negative values replacement; panel B, D, F: results obtained from Albers' model with 10%, 50% and 150%, respectively, background levels with negative values replacement. [file 1471-2105-10-61-S6.pdf]
