# Supplementary material for: A comparison on effects of normalisations in the detection of differentially expressed genes
Source: BMC Bioinformatics. 2009 Feb 13;10:61. doi: 10.1186/1471-2105-10-61 (PMC2680204; doi:10.1186/1471-2105-10-61)
Supplement: Additional file 5 — Table S5. Area Under the Curve (AUC) of specificity and sensitivity of the moderated t-test EBayes after the normalisations, for Albers' model with increasing percentage of background level with and without replacing negative values. For each simulated scenario, ranking of the normalisations according to the AUC is reported. [file 1471-2105-10-61-S5.pdf]

Table S2

Area Under the Curve (AUC) of Specificity and Sensitivity of the moderated t-test after the normalizations, for Albers' model with increasing percentage of background level with respect to expression level with and without replacing negative values. For each simulated scenario is also reported the ranking of the normalizations according to the AUC: the bigger the rank, the better the normalization.

	10% bg		10% bg replaced		50% bg		50% bg replaced		150% bg		150% bg replaced	
normalization	AUC	rank	AUC	rank	AUC	rank	AUC	rank	AUC	rank	AUC	rank
Raw	0.91	1	0.91	1	0.84	1	0.86	1	0.67	1	0.74	2
Global	0.91	1	0.91	1	0.84	1	0.86	1	0.71	3	0.76	4
GLOG	0.95	3	0.95	3	0.92	8	0.91	6	0.82	10	0.7	1
Lowess	0.97	5	0.97	4	0.88	3	0.9	4	0.7	2	0.75	3
P-Lowess	0.97	5	0.97	4	0.9	5	0.92	7	0.73	5	0.78	5
NeuralNet	0.96	4	0.97	4	0.9	5	0.92	7	0.72	4	0.78	5
OLIN	0.97	5	0.97	4	0.89	4	0.89	3	0.73	5	0.78	5
OSLIN	0.97	5	0.97	4	0.9	5	0.9	4	0.73	5	0.79	8
qsplineR	0.97	5	0.97	4	0.94	9	0.92	7	0.77	8	0.79	8
qsplineG	0.97	5	0.97	4	0.94	9	0.92	7	0.77	8	0.79	8
