# Supplementary material for: A comparison on effects of normalisations in the detection of differentially expressed genes
Source: BMC Bioinformatics. 2009 Feb 13;10:61. doi: 10.1186/1471-2105-10-61 (PMC2680204; doi:10.1186/1471-2105-10-61)
Supplement: Additional file 7 — Table S2. Parameters setting used in the Albers' simulation model. [file 1471-2105-10-61-S7.pdf]

Array number of grid rows	9
Array number of grid columns	4
Number of spots in a grid row	18
Number of spots in a grid column	18
Number of spot pins	16
Number of technical replicates	1
Number of genes (0 = max)	1000
Number of slides	15
Perform dye swaps	No
Gene expression filter	Yes
Reset gene filter for each slide	No
Mean signal	11.492
Change in log2ratio due to upregulation	0.832
Change in log2ratio due to downregulation	-0.605
Variance of gene expression	1.775
% of upregulated genes	3
% of downregulated genes	3
Correlation between channels	0.89
Dye filter	No
Reset dye filter for each slide	Yes
Channel (dye) variation	0.51
Gene x Dye	0
Error filter	yes
Reset error filter for each slide	Yes
Random noise standard deviation	0.219
Tail behaviour in the MA plot	0.11
Non-linearity filter	Yes
Reset non-linearity filter for each slide	Yes
Non-linearity parameter curvature	Yes
Non-linearity parameter tilt	0.777
Non-linearity from scanner filter	Yes
Reset non-linearity scanner filter for each slide	Yes
Scanning device bias (0 = clipped; 1 = fully non-linear)	0.295
spotpin deviation filter	Yes
Reset spotpin filter for each slide	No
Spotpin variation	0.36
Background filter	Yes
Reset background filter for each slide	Yes
Number of background densities	2
Mean standard deviation per background density	0.3
Maximum of the background signal (%) relative to the non-background signals	10, 50, 150
Standard deviation of the random noise for the background signals	0.1
Background gradient filter	Yes
Reset gradient filter for each slide	Yes
Maximum slope of the linear tilt	700
Missing values filter	Yes
Reset missing spots filter for each slide	Yes
Number of hairs	10
Maximum length of hair	20
Number of discs	6
Average radius disc	10
Number of missing spots	1000
